# Seasonal activity patterns of a Kalahari mammal community: Trade‐offs between environmental heat load and predation pressure

**DOI:** 10.1002/ece3.11304

**Published:** 2024-04-15

**Authors:** Mika M. Vermeulen, Hervé Fritz, W. Maartin Strauss, Robyn S. Hetem, Jan A. Venter

**Affiliations:** ^1^ Department of Conservation Management Nelson Mandela University George Western Cape South Africa; ^2^ Sustainability Research Unit Nelson Mandela University George Western Cape South Africa; ^3^ International Research Laboratory REHABS, CNRS – Université de Lyon 1 – Nelson Mandela University George Western Cape South Africa; ^4^ School of Biological Sciences University of Canterbury Christchurch New Zealand; ^5^ School of Animal, Plant and Environmental Sciences University of the Witwatersrand Johannesburg Gauteng South Africa

**Keywords:** anti‐predator behaviour, arid savanna, behavioural plasticity, camera traps, miniature black globe thermometers (miniglobe), thermal landscape

## Abstract

Mammals in arid zones have to trade off thermal stress, predation pressure, and time spent foraging in a complex thermal landscape. We quantified the relationship between the environmental heat load and activity of a mammal community in the hot, arid Kalahari Desert. We deployed miniature black globe thermometers within the existing Snapshot Safari camera trap grid on Tswalu Kalahari Reserve, South Africa. Using the camera traps to record species' activity throughout the 24‐h cycle, we quantified changes in the activity patterns of mammal species in relation to heat loads in their local environment. We compared the heat load during which species were active between two sites with differing predator guilds, one where lion (*Panthera leo*) biomass dominated the carnivore guild and the other where lions were absent. In the presence of lion, prey species were generally active under significantly higher heat loads, especially during the hot and dry spring. We suggest that increased foraging under high heat loads highlights the need to meet nutritional requirements while avoiding nocturnal activity when predatory pressures are high. Such a trade‐off may become increasingly costly under the hotter and drier conditions predicted to become more prevalent as a result of climate change within the arid and semi‐arid regions of southern Africa.

## Graphical abstract

Mammals in arid zones have to trade off thermal stress, predation pressure, and time spent foraging in a complex thermal landscape. In the presence of lion (*Panthera leo*) prey species were generally active under significantly higher heat loads, especially during the hot and dry spring. We suggest that increased foraging under high heat loads highlights the need to meet nutritional requirements while avoiding nocturnal activity when predatory pressures are high.

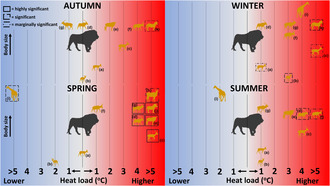

## 1 INTRODUCTION

Southern Africa's arid and semi‐arid regions will become progressively hotter and drier with climate change (Engelbrecht et al., 2015; IPCC, 2021; Van Wilgen et al., 2016). However, mammals in arid zones already face extreme climatic conditions, including high air temperatures, intense solar radiation, and low and unpredictable rainfall (Mucina & Rutherford, 2011; Schulze, 2003). When ambient temperature exceeds body temperature, mammals rely solely on evaporative cooling to dissipate heat, and in water‐scare areas, they may have to trade‐off cooling themselves evaporatively against risking hyperthermia if they are unable to replace lost body water (Cain et al., 2006; Hetem et al., 2016). They can partially alleviate this trade‐off through the selection of cooler microclimates (Van Beest et al., 2012; Mole et al., 2016) and changes in posture or orientation to reduce the heat load from direct solar radiation (Hetem et al., 2011; Maloney et al., 2005). At the landscape scale, species may even alter their use of habitat types, thereby buffering their exposure to warmer macroclimates (Srinivasan et al., 2019; Tourani et al., 2023). Another strategy includes behavioural changes, such as changes in the timing of activity, which may prevent high metabolic heat production as a result of activity coinciding with high ambient temperatures (Brivio et al., 2024; Hetem et al., 2012). For example, steenbok (*Raphicerus campestris*) reduce activity during the hottest times of the day throughout the year (Du Toit, 1993), while common duiker (*Sylvicapra grimmia*) (Ehlers Smith et al., 2019), impala (*Aepyceros melampus*) (Shrestha et al., 2014), and red hartebeest (*Alcelaphus buselaphus*) (Ben‐Shahar & Fairall, 1987) do this primarily during the hottest times of the year. In the hot, hyper‐arid desert of Saudi Arabia, the Arabian oryx (*Oryx leucoryx*) compensated for lost diurnal foraging time while shade‐seeking by shifting to nocturnal activity during the hot and dry summer (Hetem et al., 2012), in an environment devoid of predators. Prey species need to constantly weigh up the risks of predation while avoiding extreme temperatures and simultaneously acquiring adequate forage.

Predators in African savannas are generally most active during the cooler nocturnal and crepuscular periods (Clauss et al., 2021; Hayward & Hayward, 2007; Hayward & Slotow, 2009; Saleni et al., 2007). To reduce the overlap in activity with predators, prey species limit their activity during times of increased nocturnal predator activity (Tambling et al., 2015). While this change in behaviour reduces nocturnal levels of prey activity, it results in an increase in their activity levels during warmer conditions (Veldhuis et al., 2020). Typically, changes in behaviour are considered from a biologically important temporal perspective (Frey et al., 2017; Gilbert et al., 2022), but the resulting thermal trade‐off is often overlooked (Veldhuis et al., 2020), despite the potential physiological and behavioural consequences of heat loads that may impact fitness (Fuller et al., 2021; Weyer et al., 2020). Moreover, the thermal environment experienced by mammals differs based on their body size (Du Toit & Yetman, 2005), feeding ecology (Beale et al., 2017), and pelage characteristics (Hofmeyr, 1985). Therefore, changes in heat load and the associated fitness costs will not affect all species equally.

Forage has continual spatial–temporal changes in quality and quantity (Birkett et al., 2012; Van Beest et al., 2010; Venter et al., 2015) and when resources are scarce, herbivores are forced to spend more time searching for food to meet their nutritional demands (Owen‐Smith et al., 2010; Owen‐Smith & Goodall, 2014). For example, in times of drought, springbok (*Antidorcas marsupialis*) reduce their time resting and increase the time spent searching for food (Skinner & Louw, 1996). However, time spent searching for forage can be constrained in the presence of predators (Makin et al., 2018), and herbivores may be forced to forage when conditions are physiologically stressful (e.g., during the heat of the day), or risk compromising their nutritional needs (Barnier et al., 2014; Veldhuis et al., 2020). While herbivore populations are regulated by resource availability (bottom‐up) and predation (top‐down) (Hopcraft et al., 2010), their surrounding thermal landscape may influence their ability to behaviourally adjust in response to changes in these drivers (Mitchell et al., 2018).

The aim of our study was to investigate the effect of lion (*Panthera leo*) presence on the activity patterns of mammals under varying environmental heat loads. To achieve this aim, we used camera trap data collected on mammal species across four seasons in adjacent areas with two different predator guilds, one where lions were absent and the other where lion biomass predominated the carnivore guild. Concomitantly, the heat loads experienced by the prey species as they went about their daily routines were measured, within the field of view of the camera traps, with miniature black globe thermometers (miniglobes) (Hetem et al., 2007). We made predictions per functional group (adapted from Hempson et al., 2015) regarding predation vulnerability based on body size and the nested effect of predation risk, and predicted changes in activity patterns in response to seasonal changes in environmental heat loads (Appendix S1). Pairing heat load data with the activity patterns of ungulates, as determined by camera traps, is a novel approach to testing these hypotheses. We envisage that this approach may provide further insights into some of the finer scale trade‐offs that ungulates make (Hebblewhite & Merrill, 2009), particularly related to predation risk and high environmental heat loads.

## 2 MATERIALS AND METHODS

### 2.1 Study site

**FIGURE 1:**
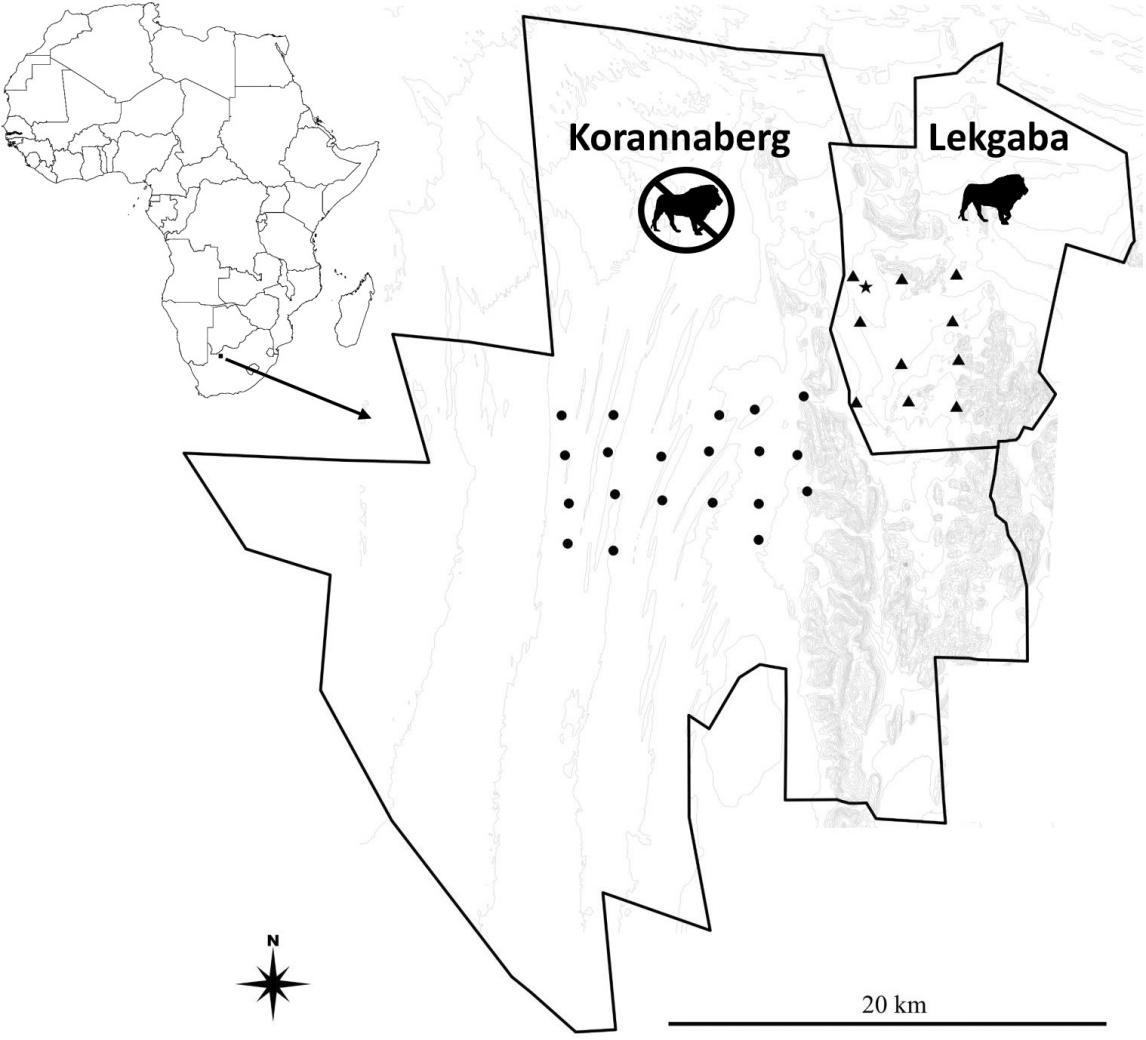
Location of Tswalu Kalahari Reserve (Tswalu) in the Northern Cape province, South Africa. Enlarged are the fenced sections of Tswalu, Korannaberg (lions absent), and Lekgaba (lions present), together with the camera traps and paired miniature black globe thermometers (circles in Korannaberg, triangles in Lekgaba). The grey contours indicate the North–South dunes and flat sandy plains in the west and the Korannaberg mountains in the East. The star indicates the location of the weather station.

We conducted our study at Tswalu Kalahari Reserve (Tswalu, 27.2031° S, 22.4673° E) in the Kalahari region of the Northern Cape, South Africa (Figure 1). The Kalahari is an arid Savanna extending through South Africa, Namibia, and Botswana (Mucina & Rutherford, 2011). The climate of the Kalahari is characterised by intense solar radiation throughout the year, exceptionally high temperatures during summer, cool winter temperatures dropping below freezing on occasion, and low and unpredictable summer rainfall (Schulze, 2003). Mean annual rainfall at Tswalu is ~325 mm (ranging between 175 and 595 mm) (Mucina & Rutherford, 2011; Van Rooyen & Van Rooyen, 2017); however, since 2014 up until the end of our study in February 2020, Tswalu experienced a severe drought (Webster & Abraham, 2021), with only half of the typical annual rain falling during our study's wet season (170 mm, summer 2019–2020, derived from Tswalu's airstrip weather station). During our study, we recorded a mean air temperature of 23°C (±10), mean maximum air temperature of 30°C (±6), and mean minimum air temperature of 15°C (±6) at Tswalu's airstrip weather station.

Tswalu is dominated by sandy plains and parallel sand dunes in the West and the Korannaberg Mountains in the East, providing an elevation gradient between 1020 and 1596 m above sea level. Tswalu is a fenced reserve and is separated into two sections, the 922 km^2^ Korannaberg section in the West and the 186 km^2^ Lekgaba section in the East (Figure 1). The land use history, habitat types, and landscape structure of both Korannaberg and Lekgaba are the same (Cromhout, 2007; Van Rooyen & Van Rooyen, 2017; Webster & Abraham, 2021). Water is provisioned throughout the year on both Korannaberg (2 waterholes/100 km^2^) and Lekgaba (3.2 waterholes/100 km^2^). While all the historical herbivore species for the region are found on both sections of Tswalu, the predator guild differs on the two sections (Webster & Abraham, 2021). On Korannaberg, the predator guild consisted of African wild dog (*Lycaon pictus*, ~4–16 adult and sub‐adult individuals, ~0.4–1.7 wild dog/100 km^2^), cheetah (*Acinonyx jubatus*, ~35–40 adult and sub‐adult individuals, ~3.8–4.3 cheetah/100 km^2^), and leopard (*Panthera pardus*, ~5–8 adult and sub‐adult individuals, ~0.5–0.9 leopard/100 km^2^). Spotted hyaena (*Crocuta crocuta*, 4 individuals, ~0.5 adult individuals/100 km^2^) were reintroduced in January 2020, the last month of our study. On Lekgaba, the predator guild consisted of lions (*Panthera leo*, ~10–18 adult and sub‐adult individuals, ~5.4–9.7 lions/100 km^2^) and leopards (~1 individual, ~0.5 leopards/100 km^2^) (Webster & Abraham, 2021). Differences in the predator guild between the two sections of Tswalu provided an opportunity for a semi‐experimental approach where we could compare prey activity in relation to the environmental heat load, on two neighbouring sites where predation risk differed. The most striking difference between the two sections of Tswalu was the dominance of lions in the biomass of predators in Lekgaba, not only making the predator biomass four times higher in Lekgaba than Korannaberg but also increasing predation pressure of large herbivore prey (Hopcraft et al., 2010).

### 2.2 Activity and temperature measurements

We deployed camera traps (Cuddeback's Professional Series White flash camera, Cuddeback, De Pere, Wisconsin, USA), which did not record any measure of temperature, in a grid formation (5 km^2^ per grid cell) on game trails (*n* = 20 on Korannaberg and *n* = 10 on Lekgaba, Figure 1). Due to camera trap shortage, and to minimise deviation from the Snapshot Safari deployment protocol of 20 camera traps per site (Pardo et al., 2021), there was an unequal deployment of camera traps between Korannaberg and Lekgaba.

In February 2019, we deployed miniature black globe thermometers (miniglobes) at 29 of the 30 Snapshot Safari camera traps at Tswalu. A miniglobe is a small hollow copper sphere (30 mm diameter, Press Spinning and Stamping Co (Pty) Ltd, Cape Town, South Africa) painted matt black, with a temperature‐sensitive data logger (iButton DS1922L, Maxim Integrated, San Jose, California, USA) inside. The data loggers were calibrated against a certified, high accuracy thermometer (Quat 100, Heraeus, Hanau, Germany) in an insulated water bath and had a resolution of 0.5°C. These miniglobes provided an integrated measure of the main components of environmental heat load, including air temperature, solar radiation, and wind speed (Hetem et al., 2007), at 30‐min intervals for one full year, until February 2020. Miniglobes were attached to wooden posts staked into the ground and placed in the open at a height of 1 m within field of view of the camera trap, thereby exposing it to the prevailing microclimatic conditions that a medium to large mammal would experience as it moves across that specific point in the landscape. The camera traps and miniglobes were serviced simultaneously and their data downloaded at 3‐month intervals.

### 2.3 Data analysis

To ensure the camera trap triggers were independent of one another, we used hourly independent data when the same species was recorded at the same camera trap (Bowkett et al., 2008; Caravaggi et al., 2018; Cusack et al., 2015; Tobler et al., 2008; Vallejo‐Vargas et al., 2022). Further, by ensuring 1‐h independence of the camera trap data, we reduced the potential for the group size of gregarious species to overinflate their levels of activity compared to those of the solitary species. We used a minimum sample size of 10 independent triggers per season for the species to be included in further analysis (Table 1) (Delibes‐Mateos et al., 2014; Fancourt et al., 2015; Gerber et al., 2012).

**TABLE 1:** The number of independent camera trap triggers for each species, during the four austral seasons, on Korannaberg (K) and Lekgaba (L).

Species	Autumn		Winter		Spring		Summer	
	K	L	K	L	K	L	K	L
Blue wildebeest (*Connochaetes taurinus*)	413	20	162	0	122	18	147	23
Common duiker (*Sylvicapra grimmia*)	126	257	108	316	56	160	51	267
Eland (*Tragelaphus oryx*)	183	0	124	0	93	12	111	0
Gemsbok (*Oryx gazella*)	701	100	460	81	297	98	244	60
Giraffe (*Giraffa camelopardalis*)	120	0	124	12	93	25	94	20
Impala (*Aepyceros melampus*)	99	34	39	21	30	36	40	21
Kudu (*Tragelaphus strepsiceros*)	186	27	93	52	90	68	123	22
Mountain zebra (*Equus zebra*)	46	0	0	0	15	13	40	0
Plains zebra (*Equus quagga*)	45	32	28	59	0	76	0	34
Red hartebeest (*Alcelaphus buselaphus*)	79	16	19	26	14	28	15	31
Steenbok (*Raphicerus campestris*)	89	62	63	104	77	57	86	52

We paired independent camera triggers to a corresponding environmental heat load recorded by the miniglobes. We assumed that if a mammal triggered a camera trap, it was active and therefore exposed to the heat load conditions measured by the associated miniglobe as it moved across that point in the landscape. Animals knocked open five miniglobes on Korannaberg and one on Lekgaba during our study, which were replaced at the following service. Because the miniglobes were visible from the camera trap, we could determine when the miniglobe was knocked down and discard all ground heat load measurements. An additional six miniglobes were lost and not replaced (four on Korannaberg and two on Lekgaba). Where a camera was not paired with a miniglobe we used the heat load data from the nearest miniglobe deployed in the most similar vegetation structure, accounting for 14% of all paired heat load records and camera triggers.

We analysed data over four austral seasons, autumn (March–May 2019), winter (June–August 2019), spring (September–November 2019), and summer (21–28 February 2019, December 2019, January–February 2020). Unfortunately, we did not collect enough camera photographs to determine lion activity to directly compare it to that of their prey under similar environmental heat loads. Therefore, we restricted our analysis to comparing the activity of prey species on a site where lions were absent (Korannaberg) and a site where lions were present (Lekgaba).

Our camera trap grids on Korannaberg and Lekgaba captured a sufficient number of photographs to compare the activity patterns of 11 different species across sites (Appendix S2). Of these, we compared the activity patterns between the two sites for eight species in autumn, winter, and summer, and 10 species in spring. To compare heat loads at which the different species were active between sites (lions present or absent) and seasons, we ran Generalised Linear Models (GLM) with a Gaussian distribution per species. For each species, our response variable was the heat load corresponding to the independent camera triggers when the species was photographed. Site (Korannaberg, lions absent and Lekgaba, lions present), season (autumn, winter, spring, and summer), and their interaction were the predictor variables, with coefficients estimating differences in heat loads corresponding to times of activity. Each model was inspected for the correct distribution of residuals using the quantile‐quantile (QQ) plots and, where necessary, heat load data was square root transformed to improve model fit (Glover‐Kapfer et al., 2019; Suraci et al., 2016; Young et al., 2020). When the analysis was done on square root‐transformed data, it has been indicated in the results of the GLM (Appendix S2); however all figures depict the actual heat loads measured by the miniglobes. We ran Tukey's post‐hoc tests to determine the significant differences between the four seasons for each species using the R package multcomp (Bretz et al., 2008) (Appendix S2). All analyses were performed with R version 4.0.4 (R Core Team, 2022).

To visualise the environmental heat loads during which species were active in relation to prevailing environmental heat loads per site, we created density plots of the distribution of these two variables using heat load measured by the miniglobes. First, we plotted the distribution of heat loads that our miniglobes recorded per season on Korannaberg and Lekgaba. Secondly, we overlaid the distribution of the heat loads recorded when a species triggered a camera trap, for the corresponding season and site, and calculated the percentage overlap to provide an index of whether species were actively avoiding certain environmental heat loads (Figure 3). This was done for the seasons during which species were recorded using significantly different heat loads per site, as determined by the GLM analyses. All density plots were created with the ggplot2 (Wickham, 2016) package in R.

## 3 RESULTS

**FIGURE 2:**
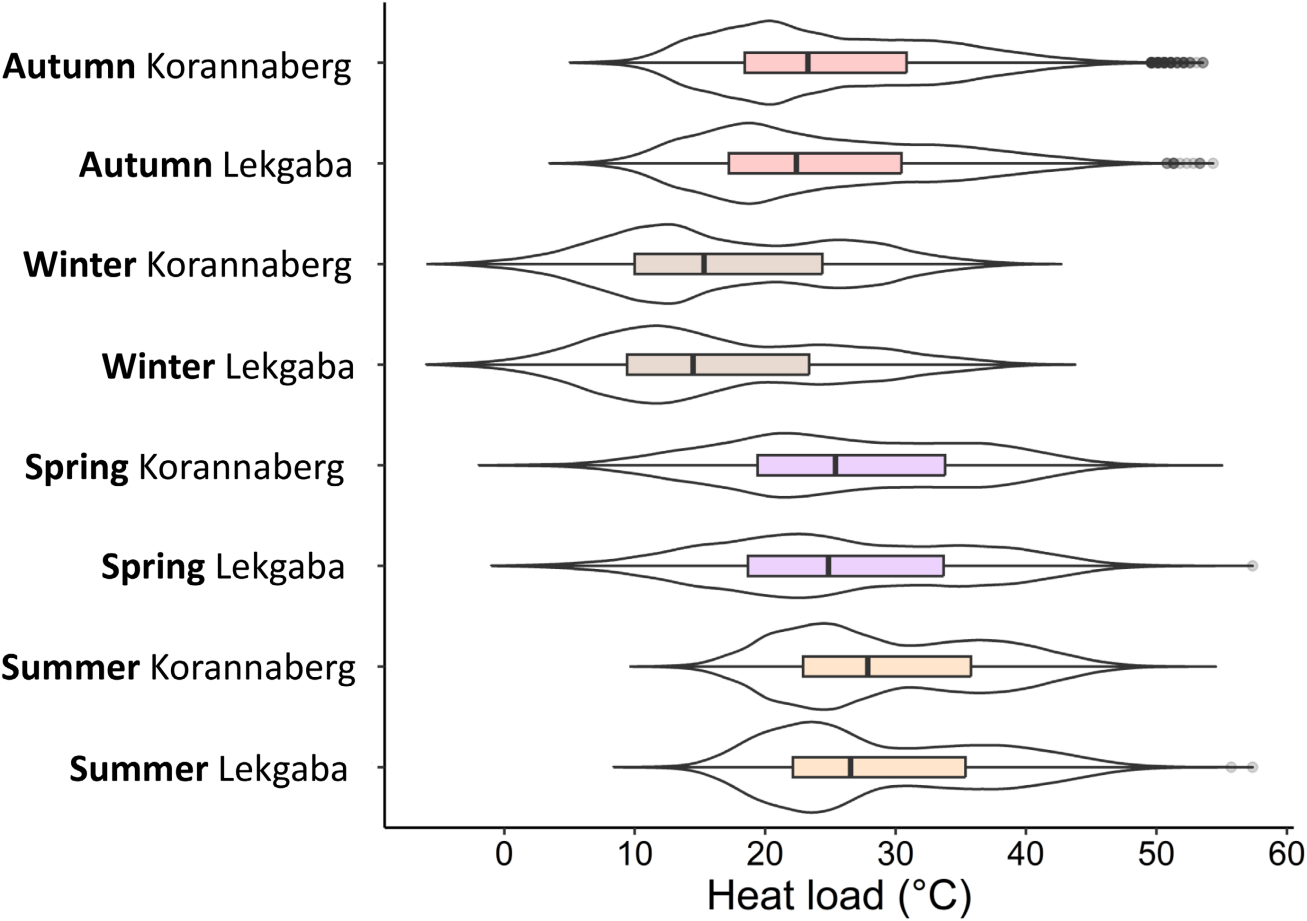
Violin plots indicating the distribution of heat loads recorded by the miniature black globe thermometers for Korannaberg (lions absent) and Lekgaba (lions present) during autumn, winter, spring, and summer, with inlaid boxplots indicating the minimum, 1st quartile, median, 3rd quartile, and maximum heat loads.

We collected 449,802 temperature records during our study period. The heat loads (median, minimum, and maximum temperatures) per season were similar between Korannaberg and Lekgaba (Figure 2). Heat loads ranged between −6 and 43°C in winter and 8 and 57°C in summer, and median heat loads in summer were 12°C warmer than in winter. Spring experienced the largest range in heat loads, with 18 days in which heat loads exceeded 50°C (Figure 2).

**FIGURE 3:**
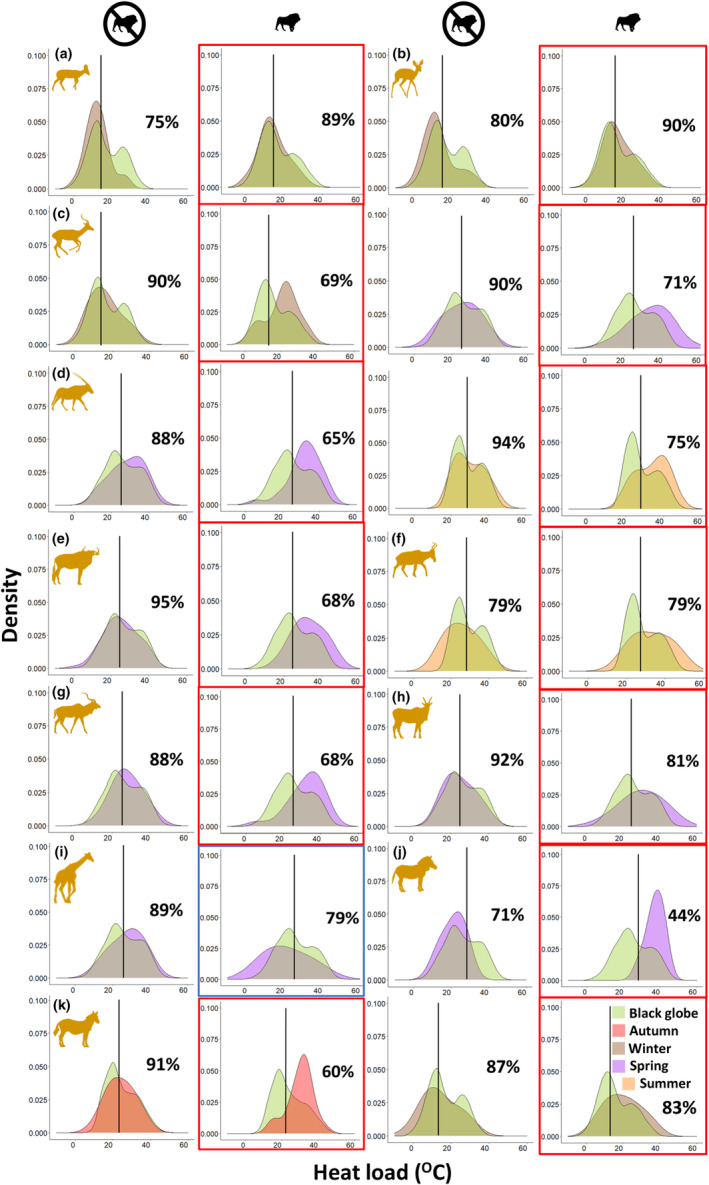
Frequency distribution of the environmental heat loads recorded by the miniature black globe thermometers per season (green) compared to the frequency distribution of the heat load corresponding to time and location of each camera trap trigger (activity) for each species per season where a significant difference in heat loads between sites was recorded (red = autumn, brown = winter, purple = spring, and orange = summer). Also included is the percentage overlap of the two frequency distributions, that is, how similar the prevailing environmental heat loads were to the heat loads during which the species was active in that season, with a lower percentage overlap suggesting stricter avoidance of activity at times coinciding with predator activity. The black vertical lines represent the median environmental heat load for each season. Plots highlighted with a red or blue border highlight the instance where species were active at significantly higher (red) or lower (blue) heat loads on the site where lions were present (Lekgaba) compared to the site where lions were absent (Korannaberg), as indicated by our GLM analyses (see Appendix S2 for details). (a) common duiker (*Sylvicapra grimmia*) during winter, (b) steenbok (*Raphicerus campestris*) during winter, (c) impala (*Aepyceros melampus*) during winter and spring, (d) gemsbok (*Oryx gazella*) during spring and summer, (e) blue wildebeest (*Connochaetes taurinus*) during spring, (f) red hartebeest (*Alcelaphus buselaphus*) during summer, (g) kudu (*Tragelaphus strepsiceros*) during spring, (h) eland (*Tragelaphus oryx*) during spring, (i) giraffe (*Giraffa camelopardalis*) during spring, (j) mountain zebra (*Equus zebra*) during spring, and (k) plains zebra (*Equus quagga*) during autumn and winter. Nomenclature follows the IUCN (2022).

**FIGURE 4:**
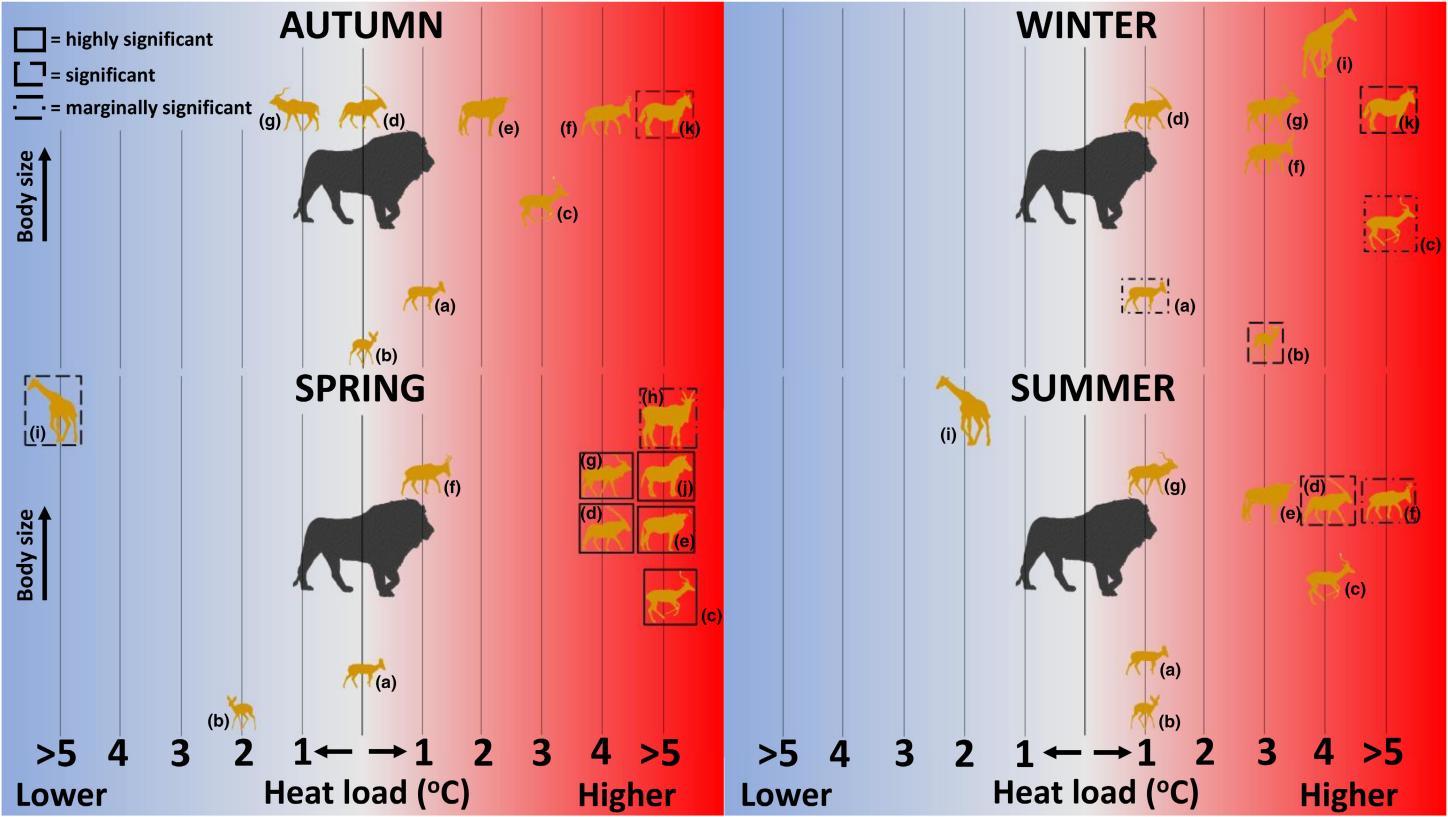
The difference in the average heat load recorded at times when mammals were active on the site where lions were present (Lekgaba) compared to the site where lions were absent (Korannaberg) per season. Activity that took place during higher heat loads in the presence of lions is indicated with a red background. Species silhouettes are ordered based on increasing body size along the y‐axis. Silhouettes with borders reflect species active at significantly higher or lower heat loads in the presence of lions; solid borders represent *p* < .001, dashed borders represent *p* < .01 and dash‐dot borders represent *p* < .05. (a) common duiker (*Sylvicapra grimmia*), b) steenbok (*Raphicerus campestris*), (c) impala (*Aepyceros melampus*), (d) gemsbok (*Oryx gazella*), (e) blue wildebeest (*Connochaetes taurinus*), (f) red hartebeest (*Alcelaphus buselaphus*), (g) kudu (*Tragelaphus strepsiceros*), (h) eland (*Tragelaphus oryx*), (i) giraffe (*Giraffa camelopardalis*), (j) mountain zebra (*Equus zebra*), and (k) plains zebra (*Equus quagga*). Nomenclature follows the IUCN (2022).

The small non‐social browsers, common duiker and steenbok, were active over a range of environmental heat loads that differed seasonally, possibly reflecting the range of prevailing environmental heat loads with transitional seasons (spring and autumn) being similar. An interaction effect between the sites and seasons highlighted seasonal modulation of responses to predation pressures (Appendix S2). During winter, the overlap between heat loads at which common duiker and steenbok were active and environmental heat loads (~90%, Figure 3a,b) resulted in exposure to significantly higher heat loads (common duiker: 1.4 ± 0.7°C, *p* = .046; steenbok: 3 ± 1°C, *p* = .002, Figure 4) on the site where lions were present (Lekgaba) than the site where lions were absent (Korannaberg).

The medium‐sized mixed feeder, impala, was active at cooler environmental heat loads in winter compared to other seasons, with an interaction evident between the sites and seasons (Appendix S2). Impala maintained a consistent overlap (~90%) between the heat loads during which they were active and the seasonal environmental heat load distribution on the site where lions were absent, suggesting activity took place throughout the range of available heat loads (Figure 3c). This overlap was lower (69%–71%) on the site where lions were present (Lekgaba) than the site where lions were absent (Korannaberg, Figure 3c), resulting in exposure to higher heat loads, particularly during winter (5 ± 2°C, *p* = .019) and spring (8 ± 2°C, *p* < .001, Figure 4).

Gemsbok (*Oryx gazella*), the only large, water‐independent grazer in our study, was active over a range of environmental heat loads that differed seasonally; only summer and spring were similar, with heat loads experienced while active being nearly 15°C higher on average in summer than winter. There was an interaction between the sites and seasons (Appendix S2). Gemsbok were active over more than 88% of prevailing environmental heat loads during spring and summer on the site where lions were absent but this overlap was reduced by up to 23% on the site where lions were present (Figure 3d). Gemsbok were active during significantly higher heat loads during spring (3.7 ± 1°C, *p* < .001) and summer (3.6 ± 1.3°C, *p* = .005, Figure 4) on the site where lions were present (Lekgaba) than on the site where lions were absent (Korannaberg).

Blue wildebeest (*Connochaetes taurinus*) were active over a range of environmental heat loads that differed seasonally, except for transitional seasons when prevailing heat loads were similar, while red hartebeest were active during cooler environmental heat loads in winter compared to other seasons. Season modulated how both the water‐dependent grazers responded to differences in predation pressure between sites (Appendix S2). During spring, blue wildebeest maintained 95% overlap between the heat loads during which they were active and the seasonal environmental heat load distribution on the site where lions were absent, whereas on the site where lions were present, blue wildebeest reduced this overlap by 27% (Figure 3e). This shift in activity resulted in the exposure to significantly higher environmental heat loads on the site where lions were present, particularly during spring (7.8 ± 2°C, *p* < .001, Figure 4). Red hartebeest maintained the same percentage overlap (79%) between sites during summer; however as illustrated by the spread of the distribution (Figure 3f), they were exposed to significantly higher environmental heat loads when active on the site where lions were present (7.4 ± 2.9°C, *p* = .013, Figure 4).

Large browsers, kudu (*Tragelaphus strepsiceros*), eland (*Tragelaphus oryx*), and giraffe (*Giraffa camelopardalis*), were active at similar environmental heat loads throughout the year, except for winter when heat loads were significantly cooler. Heat loads at the time when kudu and giraffe were active differed between sites depending on the season, as evident by a significant interaction between site and season (Appendix S2). Because of the small sample of camera triggers for eland, we were only able to compare sites in spring (Appendix S2). In spring, the activity of both kudu and eland took place under significantly higher environmental heat loads on the site where lions are present (kudu (Figure 3g): 4 ± 1.4°C, *p* = .004, eland (Figure 3h): 5 ± 2.5°C, *p* = .047, Figure 4), whereas giraffe were active at significantly lower environmental heat loads (6.4 ± 2.1°C, *p* = .002, Figures 3i and 4) on the site where lions were present than the site where lions were absent.

Mountain zebra (*Equus zebra*) were active over a similar range of environmental heat loads across three seasons on Korannaberg, being active at higher heat loads in summer than spring, whereas plains zebra (*Equus quagga*) were active at cooler environmental heat loads in winter compared to other seasons (Appendix S2). Heat loads at which the zebra were active differed between sites for all seasons for which the comparison was possible. The percentage overlap of seasonal environmental heat load distribution and the distribution of heat loads during which the species was active, highlighted that mountain zebra avoided activity at high environmental heat loads, particularly in the warm spring when they were only active for approximately two‐thirds of the prevailing environmental conditions on the site where lions were absent (Figure 3j). On the site where lions were present (Lekgaba), mountain zebra were active at environmental heat loads that were 16°C higher than the site where lions were absent (Korannaberg) in spring. Plains zebra were active at heat loads corresponding to ~90% of prevailing environmental heat loads on the site where lions were absent (Figure 3k), but this overlap decreased on the site with lions, where zebra were active at significantly higher heat loads in both autumn (6 ± 2°C, *p* = .002) and winter (5.8 ± 1.9°C, *p* = .004, Figure 4).

## 4 DISCUSSION

We explored the environmental heat loads under which mammals were active on two locations with different predator guilds. We found that mammals, in all the functional groups investigated, were active at higher heat loads during one or more season on the study site where lions were present (Lekgaba), compared to the site where lions were absent (Korannaberg). This difference in activity between sites was most noticeable during spring where 6 of the 10 species (60%) were active under significantly higher heat loads in the presence of lions. We recorded low sample size of images for mountain zebra and red hartebeest, and the number of camera traps deployed was unequal between sites. Further, actual predation events were not considered a measure of predation pressure; rather, the predator biomass, which was four times greater at Lekgaba, was assumed to result in increased predation pressure at Lekgaba. Despite these limitations, we were able to show that the presence of lions impacted the thermal conditions under which prey species were active.

Preferred prey of lions (body mass range ~150–650 kg), including gemsbok, blue wildebeest, red hartebeest, kudu, eland, mountain zebra, and plains zebra (Beukes et al., 2017; Hayward & Kerley, 2005; Owen‐Smith & Mills, 2008; Radloff & du Toit, 2004), showed an increase in heat loads at which they were active in the presence of lions compared to where lions were absent. This change in behaviour is a possible response to lions generally being active during the cooler, nocturnal, and crepuscular periods (Hayward & Hayward, 2007; Hayward & Slotow, 2009; Saleni et al., 2007; Schaller, 1979). These prey species are known to be predated upon by the predators found at Korannaberg, that is, African wild dog, cheetah, and leopard (Radloff & du Toit, 2004; Sinclair et al., 2003), our results thereby suggesting that they must face even greater predation pressure at Lekgaba if they still adjusted their behaviour to such an extent. Although impala did not fall within the preferred prey body mass range, they still behaved similarly to species known to be preferred prey of lions, that is, they were active during higher heat loads in the presence of lions, suggesting they faced greater predation pressure on the site where lions were present, whereas the two small browsers, common duiker and steenbok, were generally active during similar heat loads between sites. Because of their small body size and the size‐nested predation effect (Hopcraft et al., 2010; Sinclair et al., 2003), common duiker and steenbok likely experienced similar predation pressure on both Korannaberg and Lekgaba. Giraffes, on the other end of the body size spectrum, were active under lower heat loads in spring, on the site where lions were present. Although the large body size of giraffe places them outside the preferred prey body mass range of lions, they are still preyed upon in Ruaha National Park, Tanzania (Muneza et al., 2022); however, they are not preyed upon in another arid environment, the Kgalagadi Transfrontier Park (Beukes et al., 2017). We suggest that predation was not the strongest driver of giraffe behaviour and activity on Tswalu, but rather activity patterns were likely driven by the distribution and availability of browse and the need to meet their large daily forage requirements (Du Toit & Yetman, 2005). Prey species have several ways in which to avoid predation, including altering their times of activity and avoiding areas of high predation risk (Makin et al., 2017; Tambling et al., 2015). On Tswalu, prey species alter their activity times around waterholes in the presence of lions to reduce encounter rates (Makin et al., 2017). Our results suggested that their change in behaviour in response to predation pressure occurred across the landscape and was not only restricted to the vicinity of waterholes. Further, our results quantified the higher environmental heat loads at which prey species were active in the presence of lions, as a result of shifts in the timing of activity for intermediate‐sized herbivores (100–550 kg) in the presence of lions (Tambling et al., 2015; Veldhuis et al., 2020).

The higher heat loads at which species were active were particularly evident during spring, when conditions were hot and dry, and behavioural thermoregulation may have been prioritised in the absence of predation pressures. Vegetation productivity is strongly associated with rainfall events in the Kalahari (Tokura et al., 2018), and Tswalu experienced a long‐term drought with late summer rains during our study (Webster & Abraham, 2021). The long‐term drought and late seasonal rains would have resulted in decreased vegetation cover and a delayed growing season during spring. When vegetation quality and quantity are at their lowest, the browsing antelope, kudu, and eland (Owen‐Smith, 1998; Venter et al., 2015), the mixed‐feeder, impala (Jarman & Jarman, 1973), and the grazing ungulates, gemsbok, blue wildebeest, and Cape Mountain zebra (Beekman & Prins, 1989; Forbes & Kerley, 2022; Knight, 1991), all increase their time spent searching for forage. We suggest that to maintain required food intake rates while simultaneously avoiding activity overlap with nocturnal lions, prey species on the site where lions were present were forced to extend their daytime activity during the dry spring, resulting in activity under higher environmental heat loads. Following the onset of the summer rains, high‐quality food would have become more abundant (Tokura et al., 2018), and herbivores were probably able to meet their dietary requirements without having to extend their foraging times into the hottest parts of the day.

By comparing the distribution of heat loads during which species were active in relation to prevailing heat loads, we quantified, for the first time, whether species avoided activity under certain environmental heat loads. Common duiker showed the strongest avoidance of activity at high environmental heat loads, with only 65% overlap in the heat loads at which they were active and prevailing heat loads during the hot spring and summer (Appendix S3), perhaps coinciding with their peak in late afternoon activity (Ehlers Smith et al., 2019). Similarly, mountain zebra also show a peak in late afternoon activity in summer (Forbes & Kerley, 2022), and they too had only 68% overlap in the heat loads at which they were active and prevailing heat loads (Appendix S3). Most other species in our study, steenbok (83%), impala (86%), blue wildebeest (91%), red hartebeest (79%), and eland (88%) showed some avoidance of activity under high environmental heat loads during the summer, particularly where lions were absent (Appendix S3). Conversely, gemsbok and kudu were active over the full range of prevailing heat loads, even during the hot summer months. Both gemsbok and kudu were frequently active at environmental heat loads exceeding 40°C (Appendix S3). These heat loads were substantially higher than microclimate heat loads previously recorded by identical miniglobe thermometers on collars of both species (Boyers et al., 2019; Hetem et al., 2008), and apparently contradictory to the reduced activity of gemsbok (Boyers et al., 2021) and kudu (Owen‐Smith, 1998) on hot days, particularly when it was dry. Despite the drought conditions prevalent during our study, drinking water was provisioned on both Korannaberg and Lekgaba (Webster & Abraham, 2021). Perhaps water provisioning on Tswalu allowed gemsbok and kudu to cool themselves evaporatively while foraging throughout the 24‐hr cycle, enabling them to simultaneously meet their nutritional requirements and thermoregulatory demands (Fuller et al., 2021; Mitchell et al., 2018). Indeed, for all species, sufficient access to water to replenish water lost through evaporative cooling in the heat may have afforded herbivore prey the opportunity to prioritise avoidance of predation over thermal comfort.

In summary, high predation pressure by lions forces herbivore prey to shift the timing of their activity to hotter times of day to avoid nocturnal predation (Tambling et al., 2015; Veldhuis et al., 2020). Our results confirmed and quantified the significant increase in heat load under which prey species were active in the presence of lions. Mountain zebra, for example, were active under environmental heat loads that were 16°C warmer in the presence of lions than an adjacent site where lions were absent. Activity under higher heat loads in the presence of lions was particularly prevalent during spring, when primary production was low and herbivores needed to increase foraging at a time when ambient temperatures were increasing. The herbivore prey in our study had free access to water that may have facilitated evaporative cooling and allowed them the luxury of prioritising their nutritional requirements while avoiding predation risk without the risk of increased heat stress. Such a trade‐off may become increasingly costly under the hotter and drier conditions predicted to become more prevalent as a result of climate change within the arid and semi‐arid regions of southern Africa. To better manage herbivore populations in a warmer world, we need to better understand whether (Crawford et al., 2021) or not (Brivio et al., 2024) these trade‐offs differ across demographic groups and how temporal partitioning within the predatory guild (Cozzi et al., 2012; Hayward & Slotow, 2009) may further confine foraging times.

## AUTHOR CONTRIBUTIONS

**Hervé Fritz:** Conceptualization (equal); methodology (equal); writing – review and editing (equal). **Jan A. Venter:** Conceptualization (equal); funding acquisition (equal); methodology (equal); resources (equal); writing – review and editing (equal). **W. Maartin Strauss:** Conceptualization (equal); funding acquisition (equal); methodology (equal); resources (equal); writing – review and editing (equal). **Mika M. Vermeulen:** Conceptualization (equal); data curation (equal); formal analysis (equal); funding acquisition (equal); investigation (equal); methodology (equal); visualization (equal); writing – original draft (equal); writing – review and editing (equal). **Robyn S. Hetem:** Conceptualization (equal); methodology (equal); resources (equal); writing – review and editing (equal).

## CONFLICT OF INTEREST STATEMENT

None.

## Supporting information


**Appendices S1–S3**.

## Data Availability

The data that support the findings of this study are openly available in Mendeley Data at https://data.mendeley.com/datasets/x7h2jt4dbm/3 (doi: 10.17632/x7h2jt4dbm.3).
