# Supplementary material for: Seasonal activity patterns of a Kalahari mammal community: Trade‐offs between environmental heat load and predation pressure
Source: Ecol Evol. 2024 Apr 15;14(4):e11304. doi: 10.1002/ece3.11304 (PMC11019135; doi:10.1002/ece3.11304)

Appendix S1: Predicted changes in activity patterns in response to seasonal changes in environmental heat loads of 11 mammalian species, divided by six functional types, in the presence of different predator guilds. Nomenclature follows the IUCN (2022).

| **Functional Type Classification ^a^** | **Species** | **Predicted Response** | |
| --- | --- | --- | --- |
|  |  | **Predation vulnerability ^b, c, d, e, f^** | **Change in activity pattern (i.e., limits activity time budget)** |
| Small browsers | Common duiker (*Sylvicapra grimmia*)  Steenbok (*Raphicerus campestris*) | Similar predation risk in both Korannaberg and Lekgaba | Common duiker limit activity during the hottest times of the day during spring and summer ^g^  Steenbok limit activity during the hottest times of the day throughout the year ^h^ |
| Medium-sized mixed diet | Impala (*Aepyceros melampus*) | Similar predation risk in both Korannaberg and Lekgaba | Limit activity during the hottest times of the day during summer ^i^ |
| Water independent grazer | Gemsbok (*Oryx gazella*) | Higher predation risk in Lekgaba | Limit activity during the hottest times of the day during spring and summer ^j^ |
| Water dependent grazers | Blue wildebeest (*Connochaetes taurinus*)  Red hartebeest (*Alcelaphus buselaphus*) | Higher predation risk in Lekgaba | Blue wildebeest limit activity during the hottest times of the day throughout the year ^i^  Red hartebeest limit activity during the hottest times of the day during summer ^k^ |
| Large browsers | Kudu (*Tragelaphus strepsiceros*)  Eland (*Tragelaphus oryx*)  Giraffe (*Giraffa camelopardalis*) | Higher predation risk in Lekgaba | Kudu limit activity during the hottest times of the day during autumn and summer ^l^  Eland limit activity during the hottest times of the day throughout the year ^i^  Giraffe limit activity during the hottest times of the day throughout the year ^m^ |
| Non-ruminants | Mountain zebra (*Equus zebra*)  Plains zebra (*Equus quagga*) | Higher predation risk in Lekgaba | Mountain zebra do not limit activity during the hottest times of the day in either winter or summer ^n^  Plains zebra limit activity during the hottest times of the day throughout the year ^o^ |

The mammals in our study were grouped according to functional types adapted from Hempson et al. (2015)^a^. Predation vulnerability for each species was derived from the following literature, Hopcraft et al. (2010)^b^, Owen-Smith and Mills (2008)^c^, Radloff and du Toit (2004)^d^, Sinclair et al. (2003)^e^ and Webster and Abraham (2021)^f^. Changes in activity patterns in response to seasonal changes in environmental heat loads per functional group was derived from the following literature, Ehlers Smith et al. (2019)^g^, du Toit (1993)^h^, Shrestha et al. (2014)^i^, Boyers et al. (2019)^j^, Ben-Shahar and Fairall (1987)^k^, Owen-Smith (1998)^l^, du Toit and Yetman (2005)^m^, Forbes and Kerley (2022)^n^ and Owen-Smith and Goodall (2014)^o^

Appendix S2: Results of the Generalised Linear Models (GLM) indicating the site comparisons within seasons and the Tukey’s post-hoc test indicating the seasonal comparisons within each site. The heat loads measured by miniglobe thermometers corresponding to the independent camera trigger was the response variable, and site (Korannaberg and Lekgaba), season (autumn, winter, spring and summer), and their interaction were the predictor variables in the GLM. Values in boldface for the site comparisons within season, are those for which activity took place during significantly higher or lower heat loads on the site where lions were present (Lekgaba) compared to the site where lions were absent (Korannaberg). Values in boldface for the seasonal comparison within each site, are those for which activity took place during significantly higher or lower heat loads during that first listed season compared to the second listed season. Species silhouettes (not to scale) are provided here as reference for the main paper. Nomenclature follows the IUCN (2022).

| Species | Post hoc comparison | Estimate ± SE | P value | Dispersion parameter |
| --- | --- | --- | --- | --- |
|  |  |  |  |  |
| Blue wildebeest | Autumn: Lekgaba vs Korannaberg | 1.7±1.8 | 0.343 | 63.3 |
| (*Connochaetes taurinus*) | **Spring: Lekgaba vs Korannaberg** | **7.8±2** | **< 0.001** |  |
| ~ site * season | Summer: Lekgaba vs Korannaberg | 2.8±1.8 | 0.112 |  |
| 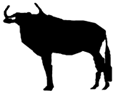 | Korannaberg: spring vs autumn | 1.6±0.8 | 0.213 |  |
|  | **Korannaberg: summer vs autumn** | **4.4±0.8** | **0.001** |  |
|  | **Korannaberg: winter vs autumn** | **-7.0±0.8** | **0.001** |  |
|  | **Korannaberg: summer vs spring** | **2.8±1** | **0.026** |  |
|  | **Korannaberg: winter vs spring** | **-8.6±1** | **0.001** |  |
|  | **Korannaberg: winter vs summer** | **-11.3±0.9** | **0.001** |  |
|  | **Lekgaba: spring vs autumn** | **7.6±2.5** | **0.007** |  |
|  | Lekgaba: summer vs autumn | 5.5±2.4 | 0.055 |  |
|  | Lekgaba: summer vs spring | -2.1±2.5 | 0.655 |  |
| Common duiker | Autumn: Lekgaba vs Korannaberg | 0.6±0.7 | 0.414 | 39 |
| (*Sylvicapra grimmia*) | **Winter: Lekgaba vs Korannaberg** | **1.4±0.7** | **0.046** |  |
| ~ site * season | Spring: Lekgaba vs Korannaberg | 0.4±1 | 0.708 |  |
| 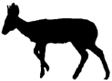 | Summer: Lekgaba vs Korannaberg | 0.7±0.9 | 0.432 |  |
|  | Korannaberg: spring vs autumn | -0.4±0.9 | 0.969 |  |
|  | **Korannaberg: summer vs autumn** | **3.1±1** | **0.006** |  |
|  | **Korannaberg: winter vs autumn** | **-7.2±0.8** | **0.001** |  |
|  | **Korannaberg: summer vs spring** | **3.6±1.1** | **0.008** |  |
|  | **Korannaberg: winter vs spring** | **-6.8±1** | **0.001** |  |
|  | **Korannaberg: winter vs summer** | **-10.3±1** | **0.001** |  |
|  | Lekgaba: spring vs autumn | -0.6±0.6 | 0.772 |  |
|  | **Lekgaba: summer vs autumn** | **3.3±0.5** | **< 0.001** |  |
|  | **Lekgaba: winter vs autumn** | **-6.3±0.5** | **< 0.001** |  |
|  | **Lekgaba: summer vs spring** | **4.0±0.6** | **< 0.001** |  |
|  | **Lekgaba: winter vs spring** | **-5.7±0.6** | **< 0.001** |  |
|  | **Lekgaba: winter vs summer** | **-9.7±0.5** | **< 0.001** |  |
| Eland | **Spring: Lekgaba vs Korannaberg** | **5±2.5** | **0.047** | 68 |
| (*Tragelaphus oryx*) | Korannaberg: spring vs autumn | 0.7±1 | 0.894 |  |
| ~ site + season | **Korannaberg: summer vs autumn** | **2.8±1** | **0.021** |  |
| 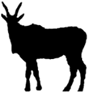 | **Korannaberg: winter vs autumn** | **-7.2±1** | **0.001** |  |
|  | Korannaberg: summer vs spring | 2.1±1.2 | 0.261 |  |
|  | **Korannaberg: winter vs spring** | **-7.9±1.1** | **0.001** |  |
|  | **Korannaberg: winter vs summer** | **-10.0±1.1** | **0.001** |  |
| Gemsbok | Autumn: Lekgaba vs Korannaberg | 0±0.9 | 0.998 | 78.4 |
| (*Oryx gazella*) | Winter: Lekgaba vs Korannaberg | 0.5±1.1 | 0.627 |  |
| ~ site * season | **Spring: Lekgaba vs Korannaberg** | **3.7±1** | **< 0.001** |  |
| 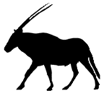 | **Summer: Lekgaba vs Korannaberg** | **3.6±1.3** | **0.005** |  |
|  | **Korannaberg: spring vs autumn** | **1.6±0.6** | **0.045** |  |
|  | **Korannaberg: summer vs autumn** | **3.4±0.7** | **0.001** |  |
|  | **Korannaberg: winter vs autumn** | **-8.0±0.5** | **0.001** |  |
|  | Korannaberg: summer vs spring | 1.8±0.8 | 0.083 |  |
|  | **Korannaberg: winter vs spring** | **-9.6±0.7** | **0.001** |  |
|  | **Korannaberg: winter vs summer** | **-11.4±0.7** | **0.001** |  |
|  | **Lekgaba: spring vs autumn** | **5.3±1.3** | **< 0.001** |  |
|  | **Lekgaba: summer vs autumn** | **7.0±1.4** | **< 0.001** |  |
|  | **Lekgaba: winter vs autumn** | **-7.5±1.3** | **< 0.001** |  |
|  | Lekgaba: summer vs spring | 1.8±1.5 | 0.617 |  |
|  | **Lekgaba: winter vs spring** | **-12.8±1.3** | **< 0.001** |  |
|  | **Lekgaba: winter vs summer** | **-14.6±1.5** | **< 0.001** |  |
| Giraffe | Winter: Lekgaba vs Korannaberg | 3.8±2.8 | 0.181 | 86.1 |
| (*Giraffa camelopardalis*) | **Spring: Lekgaba vs Korannaberg** | **-6.4±2.1** | **0.002** |  |
| ~ site * season | Summer: Lekgaba vs Korannaberg | -1.7±2.3 | 0.464 |  |
| 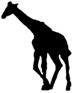 | Korannaberg: spring vs autumn | 0.5±1.2 | 0.981 |  |
|  | Korannaberg: summer vs autumn | 2.2±1.2 | 0.293 |  |
|  | **Korannaberg: winter vs autumn** | **-8.0±1.2** | **< 0.001** |  |
|  | Korannaberg: summer vs spring | 1.7±1.3 | 0.567 |  |
|  | **Korannaberg: winter vs spring** | **-8.5±1.2** | **< 0.001** |  |
|  | **Korannaberg: winter vs summer** | **-10.2±1.2** | **< 0.001** |  |
|  | Lekgaba: summer vs spring | 6.4±3.0 | 0.076 |  |
|  | Lekgaba: winter vs spring | 1.7±3.5 | 0.874 |  |
|  | Lekgaba: winter vs summer | -4.7±3.6 | 0.388 |  |
| Impala^#^ | Autumn: Lekgaba vs Korannaberg | 0.2±0.2 | 0.132 | 0.6 |
| (*Aepyceros melampus*) | **Winter: Lekgaba vs Korannaberg** | **0.5±0.2** | **0.019** |  |
| 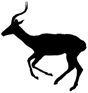~ site * season | **Spring: Lekgaba vs Korannaberg** | **0.7±0.2** | **< 0.001** |  |
|  | Summer: Lekgaba vs Korannaberg | 0.4±0.2 | 0.078 |  |
|  | Korannaberg: spring vs autumn | 0.0±0.1 | 1.000 |  |
|  | Korannaberg: summer vs autumn | 0.1±0.1 | 0.728 |  |
|  | **Korannaberg: winter vs autumn** | **-1.0±0.1** | **< 0.001** |  |
|  | Korannaberg: summer vs spring | 0.2±0.2 | 0.806 |  |
|  | **Korannaberg: winter vs spring** | **-0.9±0.2** | **< 0.001** |  |
|  | **Korannaberg: winter vs summer** | **-1.1±0.2** | **< 0.001** |  |
|  | Lekgaba: spring vs autumn | 0.5±0.2 | 0.087 |  |
|  | Lekgaba: summer vs autumn | 0.3±0.2 | 0.631 |  |
|  | **Lekgaba: winter vs autumn** | **-0.7±0.2** | **0.012** |  |
|  | Lekgaba: summer vs spring | -0.2±0.2 | 0.834 |  |
|  | **Lekgaba: winter vs spring** | **-1.2±0.2** | **0.001** |  |
|  | **Lekgaba: winter vs summer** | **-1.0±0.3** | **0.001** |  |
| Kudu | Autumn: Lekgaba vs Korannaberg | -1.4±1.8 | 0.419 | 75.4 |
| (*Tragelaphus strepsiceros*) | Winter: Lekgaba vs Korannaberg | 2.6±1.5 | 0.086 |  |
| ~ site * season | **Spring: Lekgaba vs Korannaberg** | **4±1.4** | **0.004** |  |
| 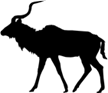 | Summer: Lekgaba vs Korannaberg | 0.8±2 | 0.705 |  |
|  | Korannaberg: spring vs autumn | -0.7±1.1 | 0.918 |  |
|  | Korannaberg: summer vs autumn | 2.2±1.0 | 0.101 |  |
|  | **Korannaberg: winter vs autumn** | **-8.0±1.1** | **0.001** |  |
|  | Korannaberg: summer vs spring | 2.9±1.2 | 0.058 |  |
|  | **Korannaberg: winter vs spring** | **-7.3±1.2** | **0.001** |  |
|  | **Korannaberg: winter vs summer** | **-10.3±1.2** | **0.001** |  |
|  | Lekgaba: spring vs autumn | 4.8±2.1 | 0.112 |  |
|  | Lekgaba: summer vs autumn | 4.4±2.7 | 0.349 |  |
|  | Lekgaba: winter vs autumn | -4.0±2.2 | 0.274 |  |
|  | Lekgaba: summer vs spring | -0.3±2.3 | 0.999 |  |
|  | **Lekgaba: winter vs spring** | **-8.8±1.7** | **0.001** |  |
|  | **Lekgaba: winter vs summer** | **-8.4±2.4** | **0.002** |  |
| Mountain zebra | **Spring: Lekgaba vs Korannaberg** | **16±2.3** | **< 0.001** | 37.4 |
| 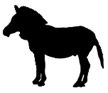(*Equus zebra*) | Korannaberg: spring vs autumn | -2.8±1.9 | 0.294 |  |
| ~ site + season | Korannaberg: summer vs autumn | 1.8±1.4 | 0.381 |  |
|  | **Korannaberg: summer vs spring** | **4.6±1.9** | **0.043** |  |
| Plains zebra | **Autumn: Lekgaba vs Korannaberg** | **6±2** | **0.002** | 71.8 |
| (*Equus quagga*) | **Winter: Lekgaba vs Korannaberg** | **5.8±1.9** | **0.004** |  |
| ~ site * season | **Korannaberg: winter vs autumn** | **-10.0±2.0** | **< 0.001** |  |
| 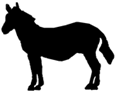 | Lekgaba: spring vs autumn | 1.3±1.8 | 0.872 |  |
|  | Lekgaba: summer vs autumn | 1.9±2.0 | 0.776 |  |
|  | **Lekgaba: winter vs autumn** | **-10.3±1.8** | **< 0.001** |  |
|  | Lekgaba: summer vs spring | 0.6±1.7 | 0.984 |  |
|  | **Lekgaba: winter vs spring** | **-11.6±1.4** | **< 0.001** |  |
|  | **Lekgaba: winter vs summer** | **-12.2±1.8** | **< 0.001** |  |
| Red hartebeest | Autumn: Lekgaba vs Korannaberg | 4.4±2.6 | 0.09 | 87.1 |
| (*Alcelaphus buselaphus*) | Winter: Lekgaba vs Korannaberg | 2.9±2.8 | 0.305 |  |
| ~ site * season | Spring: Lekgaba vs Korannaberg | 1.3±3.1 | 0.669 |  |
| 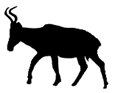 | **Summer: Lekgaba vs Korannaberg** | **7.4±2.9** | **0.013** |  |
|  | Korannaberg: spring vs autumn | 3.7±2.4 | 0.387 |  |
|  | Korannaberg: summer vs autumn | 2.4±2.3 | 0.722 |  |
|  | **Korannaberg: winter vs autumn** | **-9.0±2.1** | **0.001** |  |
|  | Korannaberg: summer vs spring | -1.4±3.1 | 0.970 |  |
|  | **Korannaberg: winter vs spring** | **-12.7±2.9** | **0.001** |  |
|  | **Korannaberg: winter vs summer** | **-11.4±2.8** | **0.001** |  |
|  | Lekgaba: spring vs autumn | 0.7±3.3 | 0.997 |  |
|  | Lekgaba: summer vs autumn | 5.4±3.3 | 0.343 |  |
|  | **Lekgaba: winter vs autumn** | **-10.5±3.4** | **0.010** |  |
|  | Lekgaba: summer vs spring | 4.7±2.8 | 0.316 |  |
|  | **Lekgaba: winter vs spring** | **-11.2±2.9** | **0.001** |  |
|  | **Lekgaba: winter vs summer** | **-15.9±2.8** | **0.001** |  |
| Steenbok^#^ | Autumn: Lekgaba vs Korannaberg | 0±0.1 | 0.853 | 0.6 |
| (*Raphicerus campestris*) | **Winter: Lekgaba vs Korannaberg** | **0.4±0.1** | **0.002** |  |
| 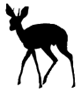~ site * season | Spring: Lekgaba vs Korannaberg | -0.2±0.1 | 0.119 |  |
|  | Summer: Lekgaba vs Korannaberg | 0.1±0.1 | 0.668 |  |
|  | Korannaberg: spring vs autumn | 2.5±1.2 | 0.170 |  |
|  | **Korannaberg: summer vs autumn** | **5.2±1.2** | **0.001** |  |
|  | **Korannaberg: winter vs autumn** | **-8.2±1.3** | **0.001** |  |
|  | Korannaberg: summer vs spring | 2.7±1.2 | 0.110 |  |
|  | **Korannaberg: winter vs spring** | **-10.6±1.3** | **0.001** |  |
|  | **Korannaberg: winter vs summer** | **-13.3±1.3** | **0.001** |  |
|  | Lekgaba: spring vs autumn | 0.1±1.4 | 1.000 |  |
|  | **Lekgaba: summer vs autumn** | **5.9±1.4** | **< 0.001** |  |
|  | **Lekgaba: winter vs autumn** | **-5.3±1.2** | **< 0.001** |  |
|  | **Lekgaba: summer vs spring** | **5.7±1.5** | **< 0.001** |  |
|  | **Lekgaba: winter vs spring** | **-5.4±1.3** | **< 0.001** |  |
|  | **Lekgaba: winter vs summer** | **-11.2±1.3** | **< 0.001** |  |

^#^Results reported for impala and steenbok were from square root transformed data.

Appendix S3: Frequency distribution of the environmental heat loads recorded by the miniature black globe thermometers per season (green) compared to the frequency distribution of the heat load corresponding to time and location of each camera trap trigger (activity) for each species per season (red = autumn, brown = winter, purple = spring and orange = summer). Also included is the percentage overlap of the two frequency distributions, i.e., how similar prevailing environmental heat loads were to heat loads during which the species was active in that season, with a lower percentage overlap suggesting avoidance of activity at times coinciding with predator activity. The black vertical lines represent the median environmental heat load for each season. Plots highlighted with a red or blue border highlight species that were active at significantly higher (red) or lower (blue) heat loads on the site where lions were present (Lekgaba) compared to the site where lions were absent (Korannaberg), as indicated by our GLM analyses (see Appendix S2 for details). A = common duiker (*Sylvicapra grimmia*), B = steenbok (*Raphicerus campestris*), C = impala (*Aepyceros melampus*), D = gemsbok (*Oryx gazella*), E = blue wildebeest (*Connochaetes taurinus*), F = red hartebeest (*Alcelaphus buselaphus*), G = kudu (*Tragelaphus strepsiceros*), H = eland (*Tragelaphus oryx*), I = giraffe (*Giraffa camelopardalis*), J = mountain zebra (*Equus zebra*) and K = plains zebra (*Equus quagga*). Nomenclature follows the IUCN (2022).


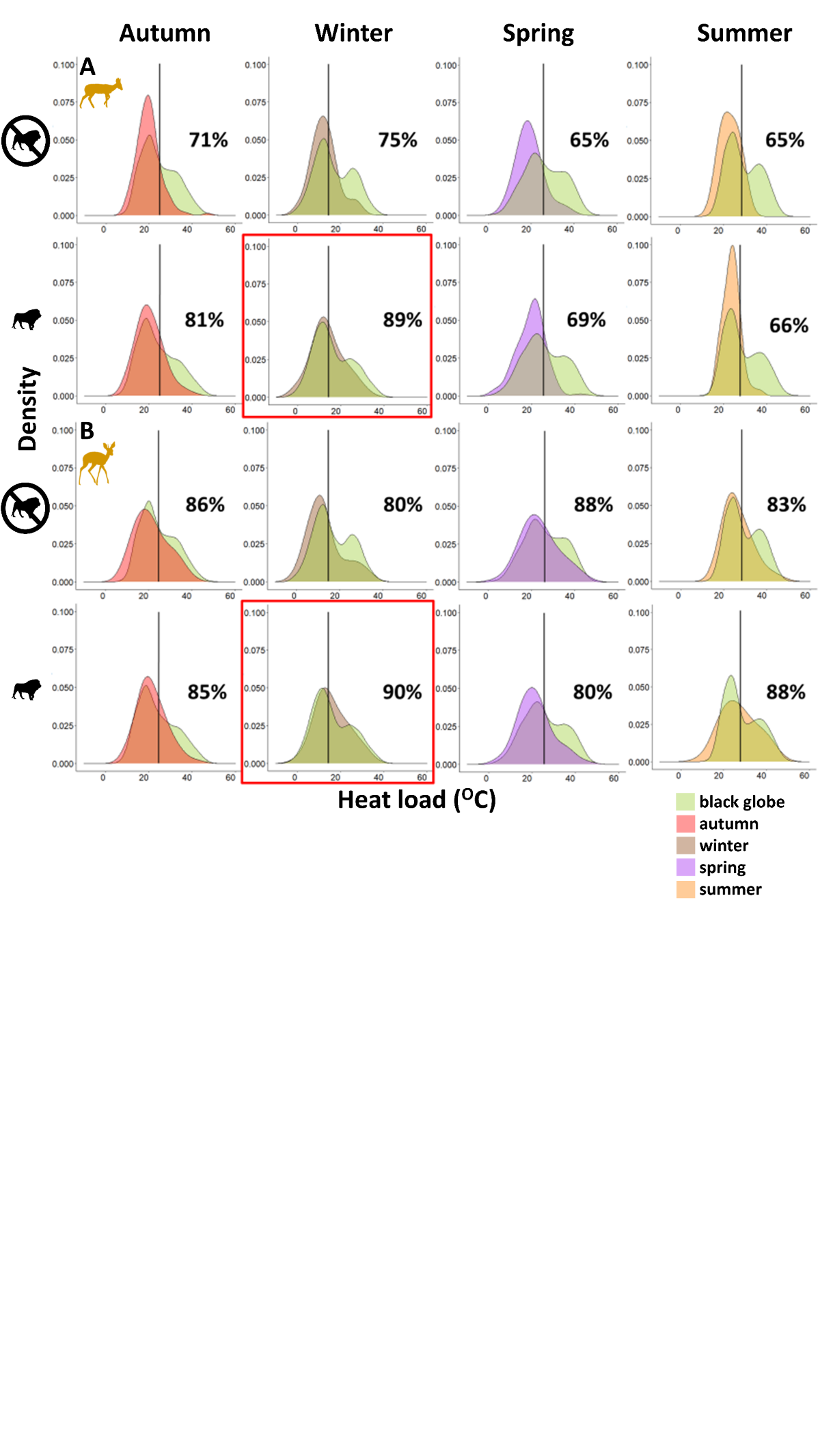


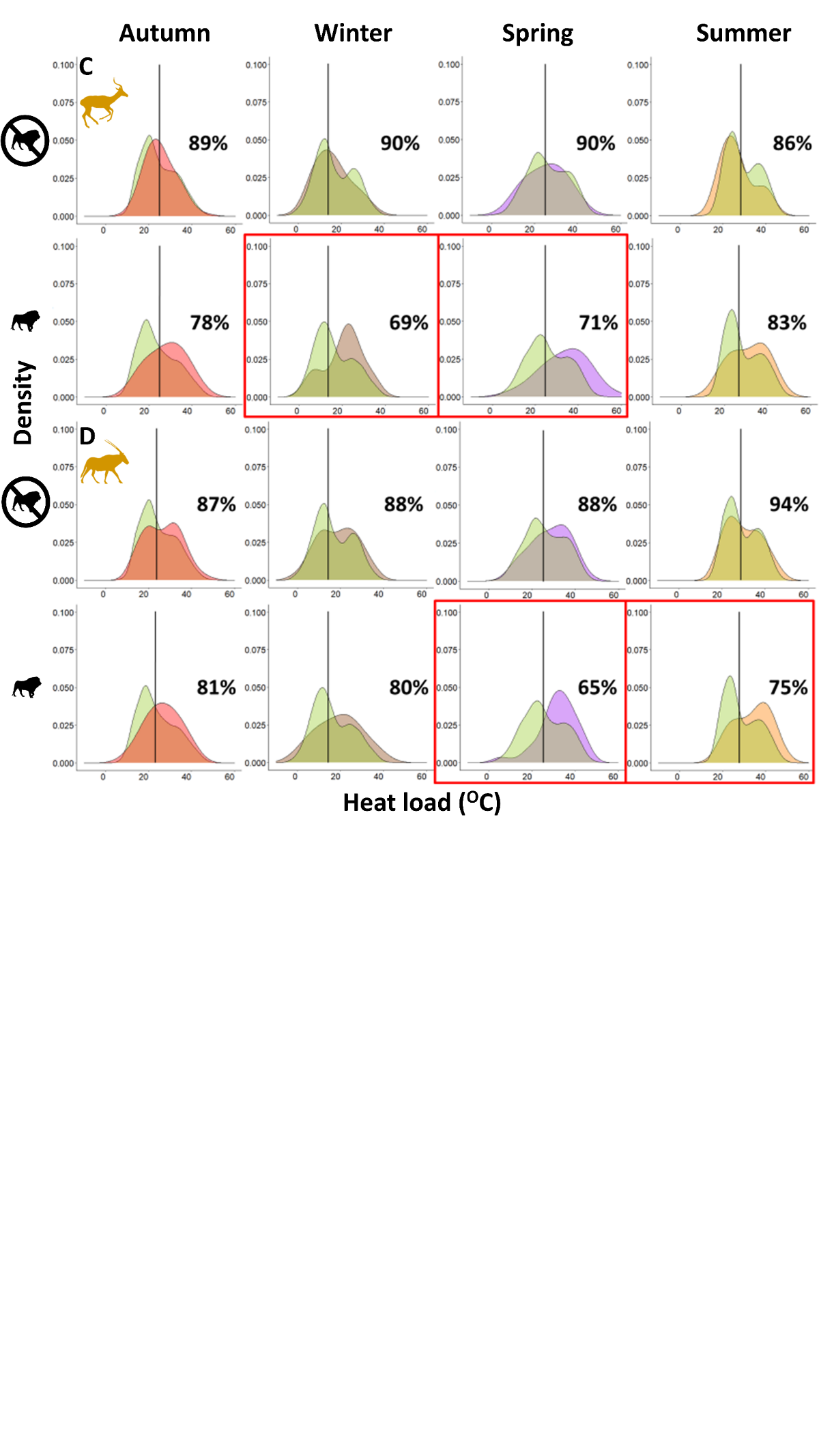


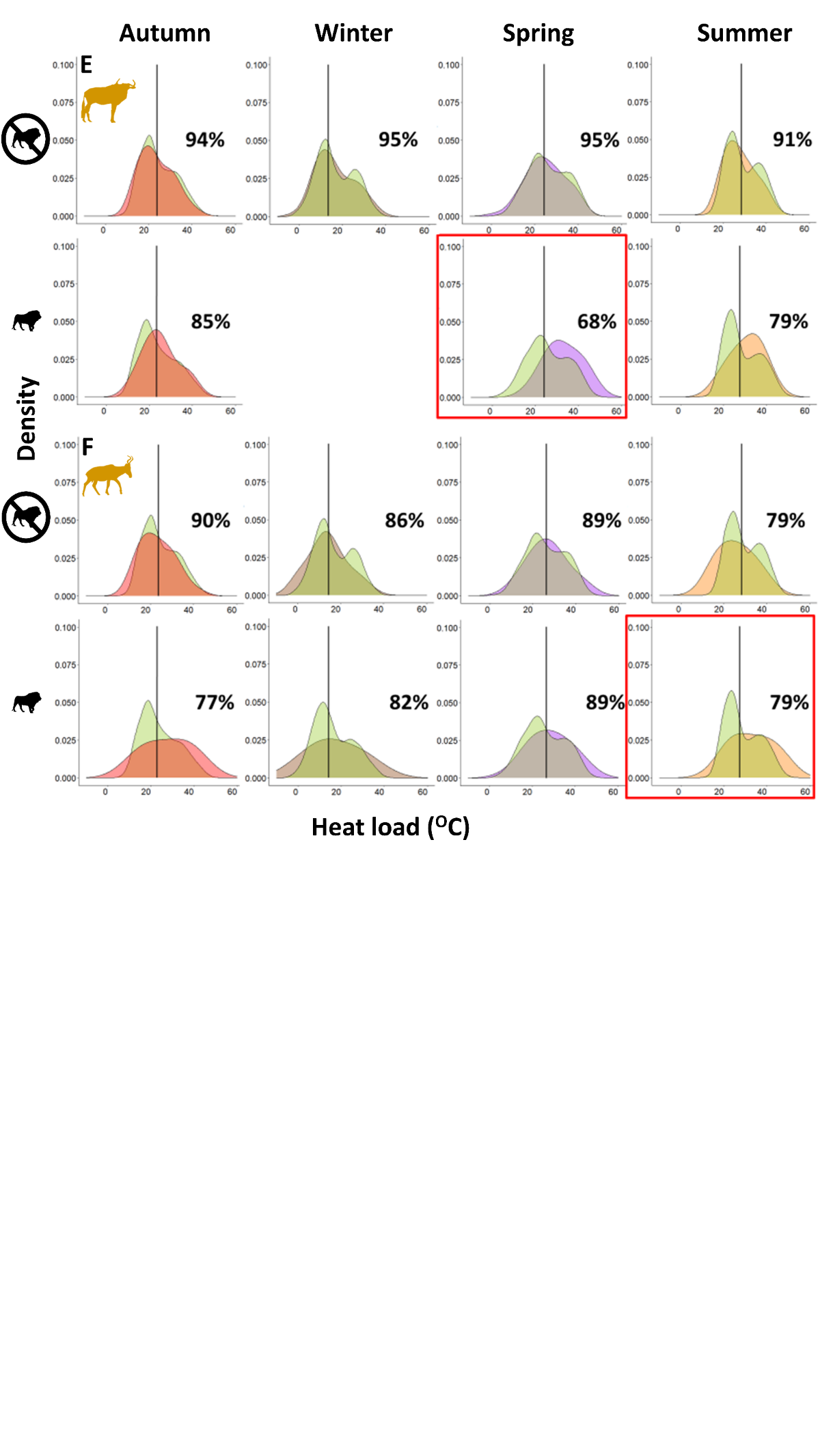


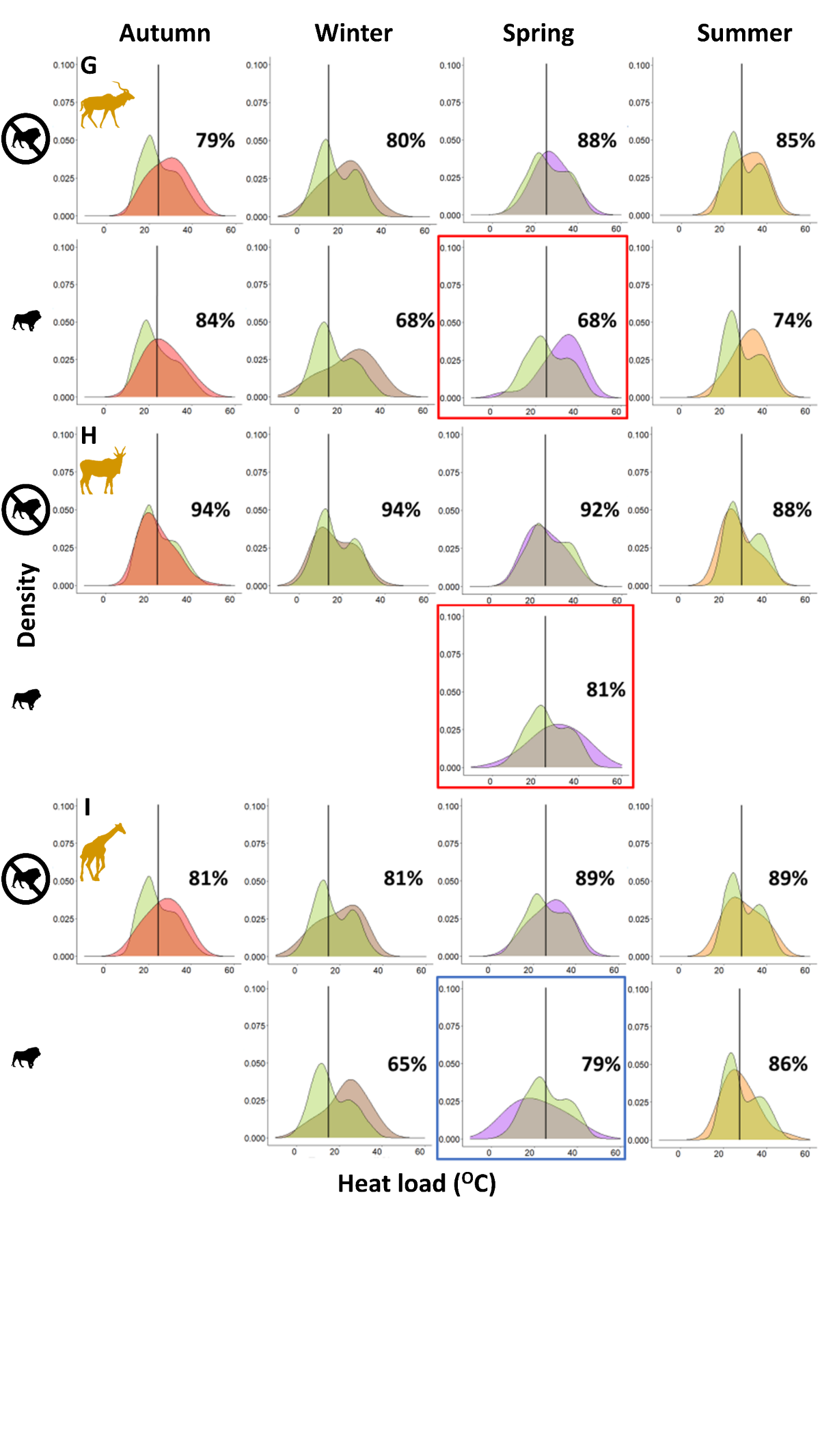


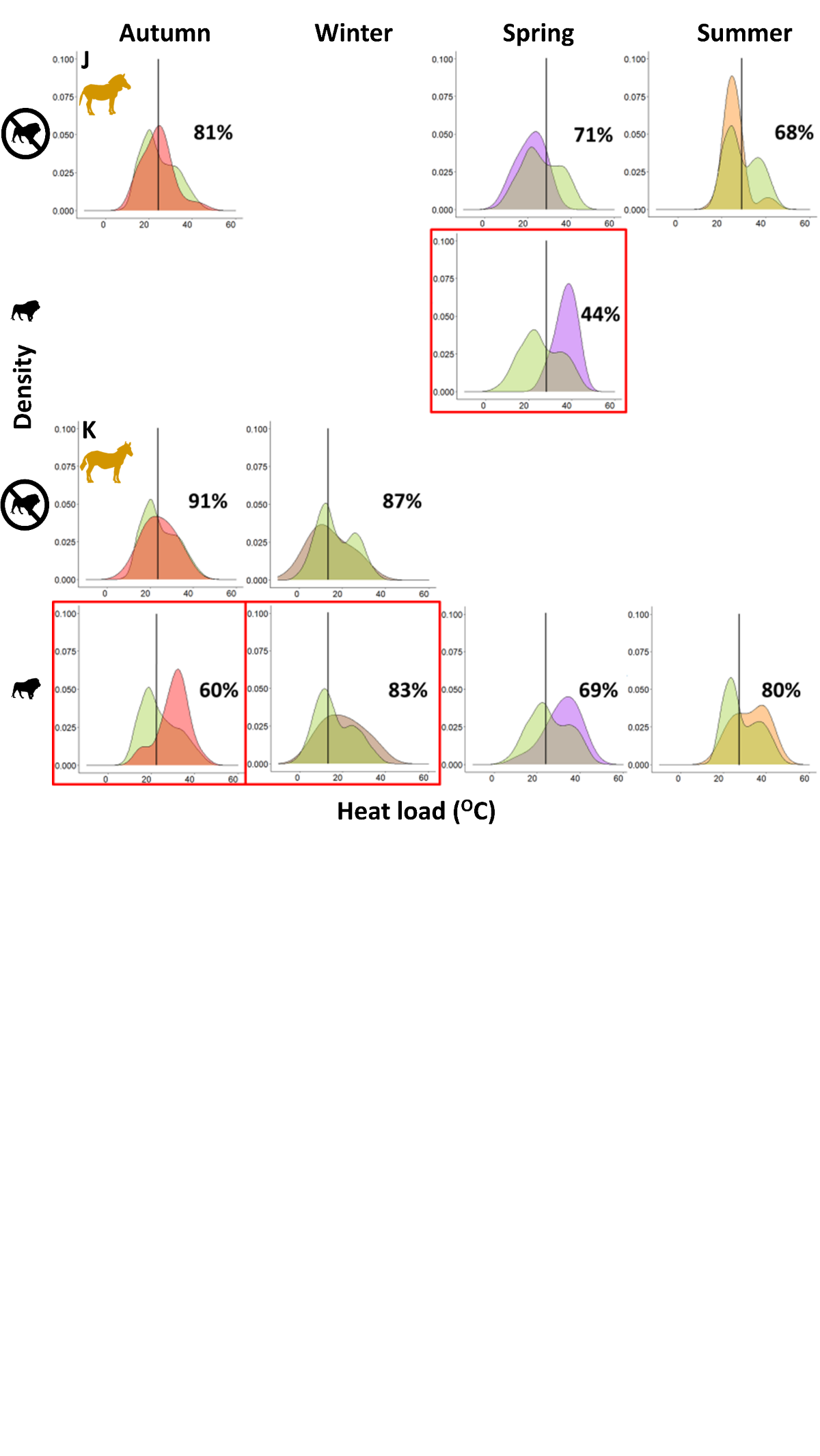

Supplement: Supplementary file 1 — Appendices S1–S3. [file ECE3-14-e11304-s001.docx]
